# Long-term safety and tolerability of eptinezumab in patients with chronic migraine: a 2-year, open-label, phase 3 trial

**DOI:** 10.1186/s12883-021-02123-w

**Published:** 2021-03-19

**Authors:** David Kudrow, Roger K. Cady, Brent Allan, Susan M. Pederson, Joe Hirman, Lahar R. Mehta, Barbara A. Schaeffler

**Affiliations:** 1 California Medical Clinic for Headache, 2001 Santa Monica Blvd, Santa Monica, CA 90404 USA; 2 Lundbeck La Jolla Research Center, 10035 Road to the Cure, Suite 250, San Diego, CA USA; 3 Alder BioPharmaceuticals, Inc. (CKA Lundbeck Seattle BioPharmaceuticals, Inc.), 11804 North Creek Parkway South, Bothell, WA USA; 4 Pacific Northwest Statistical Consulting, 18133 154th Ave NE, Woodinville, WA USA

**Keywords:** Eptinezumab, Chronic migraine, Effectiveness, Safety, Immunogenicity

## Abstract

**Background:**

Eptinezumab, an anti-calcitonin gene-related peptide monoclonal antibody recently approved in the United States for preventive treatment of migraine in adults, was found to be well tolerated in double-blind, placebo-controlled studies in patients with episodic and chronic migraine. The objective of the PREVAIL study was to evaluate the long-term safety, immunogenicity, and impact on patient-reported outcomes of repeat doses of eptinezumab in patients with chronic migraine.

**Methods:**

PREVAIL was an open-label, phase 3 trial comprising a 48-week treatment phase followed by a second 48-week treatment phase. Adults with chronic migraine received eptinezumab 300 mg by 30-min intravenous administration every 12 weeks for up to 8 doses. Patients were followed for 20 weeks after the final infusion (end-of-study visit at week 104).

**Results:**

Overall, 128 adults (mean age, 41.5 years) with chronic migraine were included. During the 2 years, the most frequently reported treatment-emergent adverse events were nasopharyngitis (14.1%), upper respiratory tract infection (7.8%), sinusitis (7.8%), influenza (6.3%), bronchitis (5.5%), and migraine (5.5%). The rate of study-drug discontinuation due to adverse events was 6.3%, which included 3 patients with infusion-related hypersensitivity. The incidence of anti-eptinezumab antibodies peaked at week 24 and declined despite continued dosing, to nondetectable levels at week 104. Improvements in patient-reported outcomes were observed at first assessment (week 4) and generally sustained through week 104.

**Conclusion:**

In adults with chronic migraine, eptinezumab 300 mg demonstrated a favorable safety profile, limited long-term immunogenicity, early and sustained reductions in migraine-related burden, and improvements in health-related quality of life over 2 years.

**Trial registration:**

ClinicalTrials.gov (Identifier: NCT02985398).

**Supplementary Information:**

The online version contains supplementary material available at 10.1186/s12883-021-02123-w.

## Background

Migraine is a chronic neurological disorder and a leading cause of long-term disability [1] and burden [2]. It is estimated that almost 40% of patients with migraine are candidates for preventive migraine treatment [3], and many patients who do receive preventive treatment discontinue due to reasons such as lack of efficacy and side effects [4–6]. Given the long-lasting nature of migraine, patients may use preventive treatment for years, underscoring the need for understanding the safety and tolerability of effective long-term migraine preventive treatment.

Eptinezumab was recently approved in the United States for the preventive treatment of migraine in adults and is a humanized monoclonal antibody that rapidly engages and inactivates calcitonin gene-related peptide (CGRP) [7], which plays a key role in initiating and mediating migraine [8–10]. In double-blind, placebo-controlled studies conducted in patients with episodic [11] and chronic migraine (CM) [12], eptinezumab was well tolerated across all doses, with no apparent dose-related trend in the nature, frequency, or severity of treatment-emergent adverse events (TEAEs).

The PREVAIL study evaluated the long-term safety of repeat doses of eptinezumab in patients with CM, as well as the pharmacokinetics, immunogenicity, and impact of eptinezumab on patient-reported outcomes (PROs). This report summarizes safety, immunogenicity, and PRO data from this study; pharmacokinetic results will be reported separately.

## Materials and methods

### Standard protocol approvals, registrations, and patient consents

The independent ethics committee and/or institutional review board for each site approved the study. All clinical work was conducted in compliance with current Good Clinical Practices as referenced in the International Conference on Harmonisation of Technical Requirements for Registration of Pharmaceuticals for Human Use guideline E6, local regulatory requirements, and the principles of the Declaration of Helsinki. All patients provided written informed consent prior to participation. PREVAIL is registered on ClinicalTrials.gov (identifier: NCT02985398; registered 07/12/2016; a list of the trial sites can be found at https://clinicaltrials.gov/ and under *Ethics approval*).

### Study design and patients

This 2-year, open-label, phase 3 trial was conducted at 20 sites in the United States from December 12, 2016, to March 7, 2019. It comprised two 48-week treatment phases, with the second phase open to those who completed the first.

Inclusion and exclusion criteria are detailed in Appendix A. Briefly, adults (18–65 years, inclusive) with a diagnosis of migraine per the International Classification of Headache Disorders 3rd edition beta (ICHD-3β, 2013, Section 1.3) [13] at or before the age of 50 years were eligible for participation if they had a history of CM for ≥12 months prior to screening and had been prescribed or recommended by a healthcare professional to use prescription or over-the-counter medication for acute and/or prophylactic migraine treatment. Individuals were excluded if they had a history or diagnosis of a headache or migraine disorder not meeting the ICHD-3β criteria for CM, required botulinum toxin injections for any medical/cosmetic reasons within 4 months prior to screening, or received any monoclonal antibody targeting the CGRP pathway within 6 months prior to screening. The study excluded patients with pre-existing significant cardiovascular disease.

### Study procedures

PREVAIL was initiated as a 1-year study with a 48-week treatment period and an end-of-study/early withdrawal visit at week 56. The protocol was modified within the first year after initiation to include a second 48-week treatment period, with an end-of-study/early withdrawal visit 20 weeks after the final dose (week 104). The total study duration was 106 weeks (including the 2-week screening period), with 12 scheduled visits (day 0 and weeks 2, 4, 8, 12, 24, 36, 48, 60, 72, 84, and 104). During the first treatment phase, patients received up to 4 doses of eptinezumab 300 mg (day 0 and weeks 12, 24, and 36). Patients who received all 4 doses in the first treatment phase (112/128 [87.5%]) could enter the second, during which they received up to 4 additional doses of eptinezumab 300 mg (weeks 48, 60, 72, and 84). Each dose was reconstituted in a total volume of 100 mL 0.9% saline and administered IV over 30 (+ 15) minutes, with infusions able to be administered for a total duration of up to 1 h, if needed, in the judgment of the investigator. Patients were monitored for at least 2 h after administration.

### Outcome measures

Safety was assessed via monitoring of TEAEs, clinical laboratory tests, physical examinations, vital signs measurements, 12-lead electrocardiograms (ECGs), and the Columbia-Suicide Severity Rating Scale (C-SSRS) [14]. TEAEs could be reported by the patient, a caregiver, the investigative site through open-ended questioning, physical examination, laboratory testing, medical records, or by other means. Per Good Clinical Practice guidelines, a TEAE was considered serious (SAE) if it resulted in death, was life-threatening, or led to other serious complications (e.g., hospitalization, significant incapacity, birth defect, etc.). The severity of each TEAE was graded (separately from seriousness) on a scale of 1 to 5: grade 1, mild; grade 2, moderate; grade 3, severe or medically significant but not immediately life-threatening; grade 4, life-threatening; or grade 5, death.

Plasma and serum for pharmacokinetic analyses and immunogenicity, respectively, were collected on day 0 and at weeks 2, 4, 8, 12, 24, 36, 48, 72, and end-of-study visit. Immunogenicity was assessed by the development of anti-eptinezumab antibodies, characterization of anti-eptinezumab antibodies for neutralizing activity, and epitope specificity of the anti-drug antibody (ADA) response.

Patient-reported outcome measures included the Migraine Disability Assessment (MIDAS) questionnaire [15], patient-identified most bothersome symptom (MBS) associated with migraine, Patient Global Impression of Change (PGIC) [16], and 6-item Headache Impact Test (HIT-6) [17, 18]. The MIDAS questionnaire was administered on day 0, at week 12, and every 12 weeks thereafter. Patients identified their MBS at screening, and the change in that MBS was rated on day 0 and at weeks 4, 8, 12, 24, 36, and 48. PGIC was administered at weeks 4, 8, and 12, and every 12 weeks thereafter. The HIT-6 was administered at screening, on day 0, at weeks 4, 12, and every 12 weeks thereafter. All PROs were assessed prior to dosing and at the end-of-study visit.

The MIDAS questionnaire measures migraine-related disability in patients’ daily lives. It comprises 5 questions about performance over the past 3 months, with responses provided in number of days. Responses are totaled to determine the level of disability: 0–5 days, grade I (little or no disability); 6–10 days, grade II (mild disability); 11–20 days, grade III (moderate disability); 21+ days, grade IV (severe disability). The American Headache Society’s position statement of 2018 deems the clinically meaningful threshold for change in MIDAS total score as a reduction of ≥5 points (days) when baseline score is 11–20 days and as ≥30% when baseline score is >20 days [19].

At screening, patients verbally identified their MBS related to migraine. These included a wide range of migraine-associated symptoms (i.e., nausea, vomiting, sensitivity to light, sensitivity to sound, mental cloudiness, fatigue, pain with activity, mood changes, or other). A post hoc medical review was conducted to recode symptoms in the “other” category and assign them to existing or new MBS categories. At each subsequent visit, patients rated the improvement in their self-identified MBS using a 7-point scale identical to that used for PGIC.

The PGIC includes a single question concerning the patient’s impression of the change in their disease status since the start of the study. Seven responses are possible: very much improved, much improved, minimally improved, no change, minimally worse, much worse, and very much worse.

The HIT-6 measures the impact of migraine on the ability to function normally in daily life. It measures 6 items (severe pain, social limitations, role limitations, cognitive functioning [4-week recall], psychological distress [4-week recall], and vitality [4-week recall]) using a Likert-type scale of frequency (never = 6, rarely = 8, sometimes = 10, very often = 11, always = 13). Total scores can range from 36 to 78, with a 6-point decrease considered clinically meaningful in CM [20]. Life impact is categorized as severe (total score  ≥60), substantial (56–59), some (50–55), and little to none (≤49).

### Statistical methods

A sample size of 120 treated patients was planned to obtain ≥90 patients with 48 weeks of safety data, assuming that ≥75% would complete the first treatment phase. All data were captured from the patient’s first visit through the end of week 48. All patients who received ≥1 dose of study medication were included in the safety population, which was used to assess safety, tolerability, immunogenicity, and PROs.

Safety endpoints were summarized using descriptive statistics. TEAEs and medical history were coded using the Medical Dictionary for Regulatory Activities (MedDRA) v20.1. Coding to the preferred term “hypersensitivity” was based on the sponsor’s established framework for evaluating individual symptoms or symptom constellations on days of dosing.

Patient-reported outcomes were summarized using descriptive statistics based upon observed data, with no imputation for missing values. One-sample *P*-values from *t*-tests based on change from baseline (i.e., testing for a non-zero change from baseline) were performed for HIT-6 and MIDAS; alpha-control was not utilized. All analyses were conducted using SAS software (SAS Institute, Inc., Cary, NC) v9.2 or higher.

## Results

**Fig. 1:**
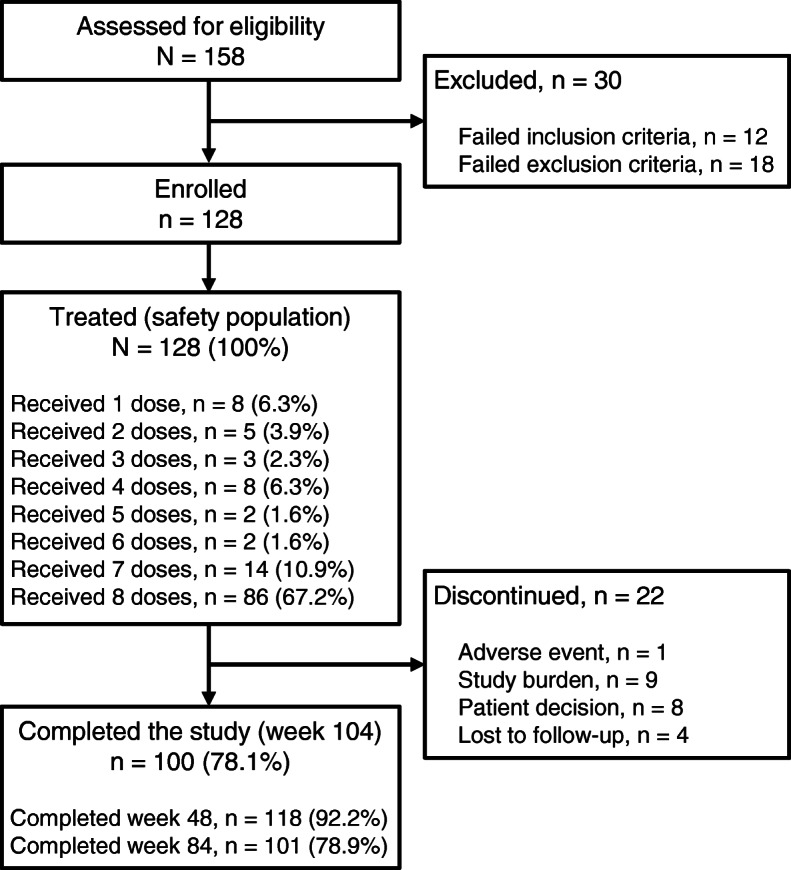
Patient disposition

All of the 128 patients enrolled received ≥1 dose of eptinezumab and were included in the safety population (Fig. 1; Table 1). Patients were predominantly female gender (85.2%), white race (95.3%), and ethnicity other than Hispanic or Latino (79.7%). The mean number of self-reported migraine and headache days per 28-day period in the 3 months prior to screening was 14.1 and 20.3, respectively; 38.3% of patients had a medication-overuse headache diagnosis (ICHD-3 criteria [21]) at baseline, which was confirmed through documented medical history and a headache questionnaire administered at screening.

**Table 1:** Demographics and baseline clinical and migraine characteristics

	Eptinezumab 300 mg *N* = 128
**Demographics**	
Mean (SD) age, years	41.5 (11.33)
Sex, n (%)	
Male	19 (14.8)
Female	109 (85.2)
Ethnicity, n (%)	
Hispanic or Latino	26 (20.3)
Not Hispanic or Latino	102 (79.7)
Race, n (%)	
White	122 (95.3)
Black or African American	4 (3.1)
Asian	1 (<1)
Multiple Races	1 (<1)
**Clinical characteristics**	
Mean (SD) weight, kg	77.8 (16.97)
Mean (SD) height, cm	166.3 (9.01)
Mean (SD) BMI, kg/m^2^	28.0 (5.07)
**Migraine history**	
Mean (SD) age at migraine diagnosis, years	20.4 (8.94)
Mean (SD) duration of migraine diagnosis, years	21.2 (11.65)
Mean (SD) number of years with CM	13.5 (11.11)
MOH diagnosis, n (%)	49 (38.3)
Mean (SD) number of headache days^a^	20.3 (3.68)
Mean (SD) number of migraine days^a^	14.1 (4.25)
Mean (SD) number of migraine attacks^a^	10.5 (4.29)
Most bothersome symptom, n (%)^b^	
Sensitivity to light	31 (24.2)
Pain	21 (16.4)
Nausea/vomiting	17 (13.3)
Pain – with activity	11 (8.6)
Sensitivity to sound	11 (8.6)
Throbbing/pulsation	8 (6.3)
Headache	4 (3.1)
Vision impacts	4 (3.1)
Cognitive disruption	5 (3.9)
Multiple	3 (2.3)
Pain – anatomical	3 (2.3)
Aura	2 (1.6)
Dizziness	2 (1.6)
Mood changes	2 (1.6)
Allodynia	1 (<1)
Eye pain	1 (<1)
Neck pain	1 (<1)
Speech difficulty	1 (<1)
**Concomitant headache medications**^c^	
Used ≥1 acute headache medication, n (%)	127 (99.2)
Thomapyrin N	57 (44.5)
Ibuprofen	52 (40.6)
Sumatriptan	43 (33.6)
Paracetamol	26 (20.3)
Naproxen sodium	13 (10.2)
Used ≥1 prophylactic medication, n (%)	46 (35.9)
Topiramate	16 (12.5)

*BMI* Body mass index, *CM* Chronic migraine, *MOH* Medication-overuse headache, *SD* Standard deviation. ^a^Self-reported average number per 28-day period in the 3 months prior to screening. ^b^Distribution of most bothersome symptom after post hoc medical review to recode “other” symptoms identified by patients at screening. ^c^Medications with a start or stop date on or after the treatment dosing date are considered concomitant

Nearly all patients (99.2%) reported using ≥1 acute concomitant headache medication and 35.9% reported using ≥1 preventive medication; the most common preventive medication was topiramate (12.5%).

### Long-term safety and tolerability

A total of 118 patients (92.2%) completed the primary treatment phase (week 48), and 101 (78.9%) completed the secondary (week 84); 100 patients (78.1%) remained 20 weeks after administration of the final study dose (week 104). The majority of patients (67.2%) received all 8 doses of eptinezumab; 87.5% received ≥4.

Overall, 91 patients (71.1%) experienced ≥1 TEAE during the study (Table 2). These events were identified during the first treatment phase of the study (by week 48) for most patients (*n* = 79). The most frequently reported TEAEs were nasopharyngitis (27 events in 18 patients), upper respiratory tract infection (18 events/10 patients), sinusitis (13 events/10 patients), influenza (11 events/8 patients), bronchitis (8 events/7 patients), and migraine (10 events/7 patients). In addition, 5 patients experienced events reported as hypersensitivity (generalized pruritus, runny nose, sneezing, wheezing, lip swelling, eye swelling, nausea, and/or vomiting). All TEAEs categorized as hypersensitivity occurred during or immediately after IV administration, were mild or moderate in severity, and resolved without treatment or routine symptom-based medical management. The occurrence of hypersensitivity TEAEs was not consistent from one infusion to the next, occurring during the first infusion in 1 patient, the second infusion in 3 patients, and the sixth infusion in 1 patient.

**Table 2:** Summary of safety and tolerability over 2 years

Event, n (%)	Eptinezumab 300 mg *N* = 128
Any TEAE	91 (71.1)
Any study drug-related TEAE	18 (14.1)
Any severe TEAE	13 (10.2)
Any serious TEAE	5 (3.9)
Any TEAE leading to study drug withdrawal	8 (6.3)
Any TEAE leading to study drug interruption	10 (7.8)
Any TEAE resulting in death	0

*TEAE* Treatment-emergent adverse event

The majority of TEAEs (95.6%) were mild or moderate in nature. Thirteen patients (10.2%) had severe TEAEs: worsening of migraine (*n* = 3 [2.3%]), and benign neoplasm, biliary dyskinesia, cholelithiasis, conversion disorder, inguinal hernia, pneumonia, tendon injury, upper respiratory tract infection, uterine leiomyoma, vision blurred, and inadequately controlled diabetes mellitus (*n* = 1 [<1%] each). All of these severe events were evaluated as not related to study drug.

Ten patients (7.8%) experienced a TEAE that led to interruption of study drug administration, the most frequently reported being infusion-site extravasation (*n* = 6; 4.7%). All incidences of infusion-site extravasation leading to interruption of study drug administration were mild in severity, considered not related to study drug, and resolved on the same day without concomitant treatment. Other TEAEs leading to interruption of study drug administration were hypersensitivity (*n* = 2), anaphylactic reaction (*n* = 1), and rhinitis (*n* = 1). Both hypersensitivity events were moderate in severity, considered related to study drug, resolved on the same day with standard symptomatic medical treatment, and led to withdrawal of treatment (see below). The anaphylaxis event was of grade 2 severity, with a serious criterion of “medically important.” This day 0 unexpected event was associated with features of an immediate type 1 (IgE-mediated) hypersensitivity event and was considered related to the study drug by the investigator. The patient’s medical history included asthma, sulfite allergy, and environmental allergies. During the TEAE, the patient had symptoms of erythema, pruritus, nasal congestion, and hives across his entire body, and began to exhibit erythema of the neck and complained of nasal stuffiness. However, the patient had no clinical manifestations of respiratory or cardiovascular compromise, which are clinical criteria required for a diagnosis of anaphylaxis [22]. Thus, this event would be more accurately described as an allergic reaction. The event was initially treated with epinephrine via injection, but there was no discernible effect within 10 min. The principal investigator then administered IV diphenhydramine, which resulted in an almost immediate response noted with reductions in itching, nasal congestion, and flushing. The rhinitis event occurred in conjunction with an allergic reaction of wheezing and swelling lips, for which the patient was withdrawn from further treatment.

Eight patients (6.3%) experienced a TEAE that led to study drug withdrawal, including 3 patients with hypersensitivity. All other events leading to withdrawal were reported for only 1 patient each (<1%; anaphylactic reaction, palpitations, infusion-site erythema, metabolism and nutrition disorders, inadequately controlled diabetes mellitus, complex regional pain syndrome, and deep vein thrombosis).

A total of 18 patients (14.1%) had ≥1 TEAE that was considered related to study drug (Table 3). The most frequently reported study-drug–related TEAEs were hypersensitivity (3.9%) and fatigue (3.1%). The remaining study-drug–related TEAEs were each reported in <1% of patients. A single patient experienced an allergic reaction following the fifth infusion, which manifested as hives on legs, itchy scalp, and lower lip swelling. Most events were mild; one patient experienced a severe event of blurred vision on day 609 that resolved within 1 day without treatment.

**Table 3:** Study drug-related treatment-emergent adverse events over 2 years

Event, n (%)	Eptinezumab 300 mg *N* = 128
Any study drug-related event	18 (14.1)
Hypersensitivity	5 (3.9)
Fatigue	4 (3.1)
Anaphylactic reaction	1 (<1)
Back pain	1 (<1)
Blood pressure systolic increased	1 (<1)
Constipation	1 (<1)
Dermatitis	1 (<1)
Dizziness	1 (<1)
Electrocardiogram ST segment depression	1 (<1)
Hypotension	1 (<1)
Influenza like illness	1 (<1)
Infusion site erythema	1 (<1)
Infusion site pruritus	1 (<1)
Lethargy	1 (<1)
Nausea	1 (<1)
Paresthesia	1 (<1)
Rhinitis	1 (<1)
Tremor	1 (<1)
Typical aura without headache	1 (<1)
Vision blurred	1 (<1)
Weight increased	1 (<1)

Five patients (3.9%) experienced a serious TEAE; only 1 was considered related to the study drug (grade 2 anaphylactic reaction, described above). No patient experienced any suicidal ideation or behavior, as determined by C-SSRS.

No clinically relevant trends in clinical laboratory, vital sign, or ECG results were identified. No patient was reported to have a QTcF interval of >500 msec or an increase in QTcF interval of >60 msec from baseline. One patient had 2 ECGs with QTcF intervals >480 msec (482 and 483 msec), both considered not clinically significant by the investigator. There was no increased rate of cardiovascular compromise observed in this study.

There was no evidence of an impact from the development of ADA, including antibodies with neutralizing potential (NAbs), on the safety profile of eptinezumab. A total of 23 patients (18%) developed antibodies to eptinezumab during the study. The incidence of ADA was maximal at 24 weeks (21/120, 17.5%), then declined despite continued dosing to 5.3% (6/113) at week 48, 4.0% (4/101) at week 72, and 0% at week 104. The incidence of NAbs among all treated patients was 7.0% (9/128). The incidence of NAbs generally increased over time from week 8 (3/7 ADA-positive patients) to week 12 (8/11 ADA-positive patients) and decreased thereafter. Of the 21 ADA-positive patients at week 24, five were NAb positive. At week 72, 0/4 ADA-positive patients were NAb positive.

There were 3 pregnancies during the study: 2 ended prematurely (1 miscarriage/1 elective termination) and 1 was carried to term (healthy baby).

### Patient-reported outcomes

**Fig. 2:**
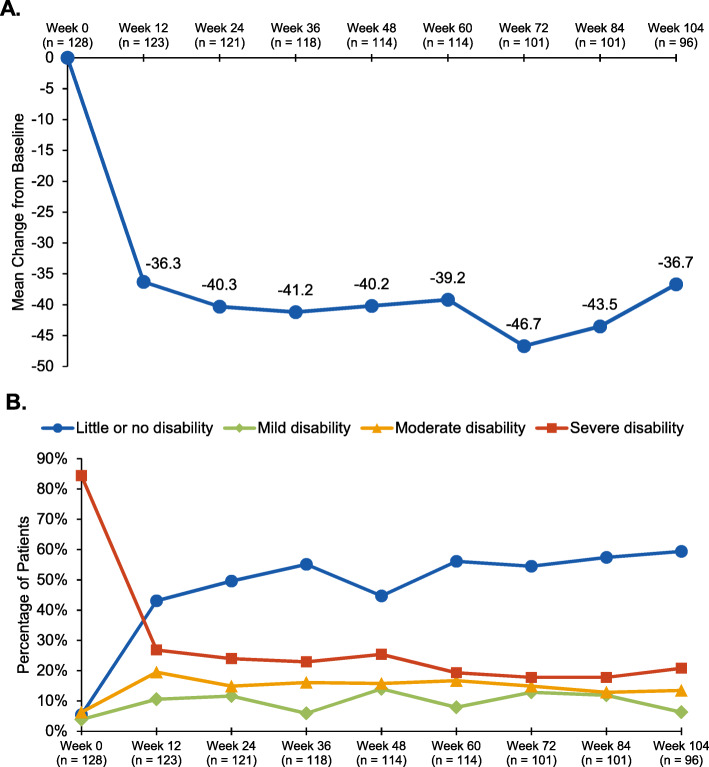
Migraine Disability Assessment total score, by visit: (**a**) mean changes from baseline and (**b**) level of disability. Mean Migraine Disability Assessment total score at baseline: 56.8. The level of disability in daily functioning was graded using the Migraine Disability Assessment total score: 0–5 days, grade I (little or no disability); 6–10 days, grade II (mild disability); 11–20 days, grade III (moderate disability); 21+ days, grade IV (severe disability) [15]

The mean (standard deviation [SD]) MIDAS total score at baseline was 56.8 (52.0) and decreased over time (Fig. 2a), starting at the first assessment (week 12), at which time it was 20.0 (40.2). At week 104, the mean (SD) MIDAS total score was 22.0 (58.9), representing a mean reduction from baseline of 36.7 (71.5). The majority of patients (84.4%) had severe disability at baseline; 5.5% had little to no disability. At week 12, the percentage of patients with severe disability was reduced to 26.8% and the percentage of patients with little to no disability increased to 43.1%. These results were generally maintained throughout the study (Fig. 2b). At week 104, the percentage of patients with severe disability was reduced to 20.8%, whereas the percentage of patients with little to no disability increased to 59.4%. Overall, the majority of patients (~ 60%) reported little or no disability or mild disability at the week-12 through week-104 assessments.

**Fig. 3:**
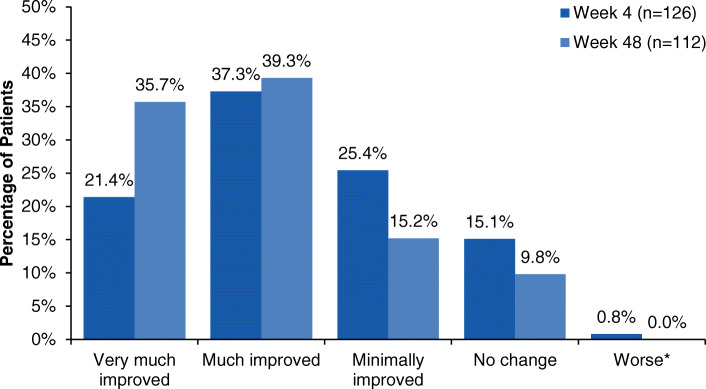
Patient-identified most bothersome symptom: change from baseline at weeks 4 and 48. *Worse includes minimally worse, much worse, and very much worse. Not collected beyond week 48

The most common patient-identified MBS at baseline was sensitivity to light, followed by pain, nausea/vomiting, pain with activity, and sensitivity to sound (Table 1). At week 4, 58.7% of patients indicated their MBS was “much improved” or “very much improved,” which increased to 75.0% by week 48 (Fig. 3). Patient-identified MBS was not collected beyond week 48.

**Fig. 4:**
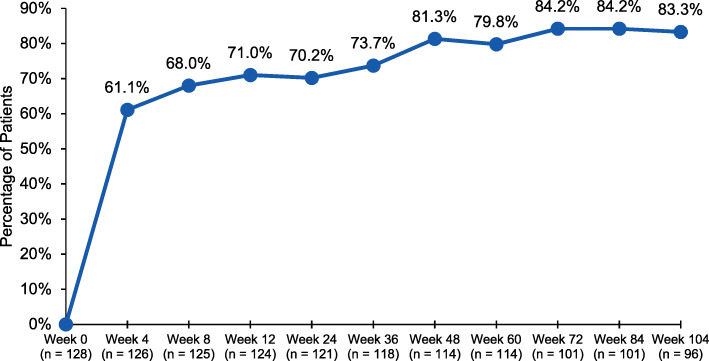
Patient Global Impression of Change: percentage of patients “much improved” or “very much improved” at each assessment

The majority of patients (61.1%) reported PGIC as “much improved” or “very much improved” at week 4 (Fig. 4). The percentages of patients reporting “much improved” or “very much improved” generally increased to 81.0% at week 48 and were maintained throughout the remainder of the study.

**Fig. 5:**
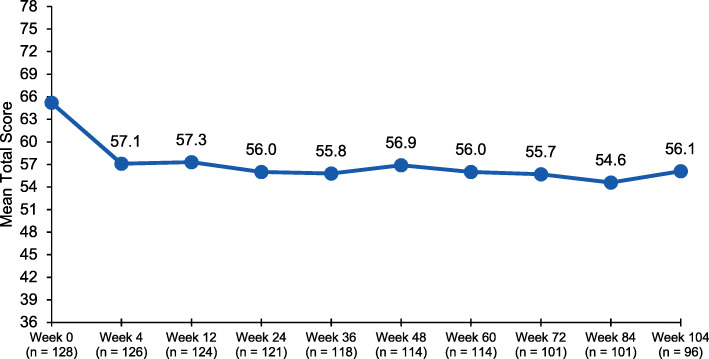
Headache Impact Test: mean total score at each assessment. Baseline: *n* = 128; mean = 65.2

Most patients (92.2%) reported severe life impact at baseline, as measured by HIT-6 total scores. The percentage of patients with severe life impact decreased to 39.7% at week 4 and ranged from 32.7 to 43.5% throughout the study, with 38.5% of patients reporting severe life impact at week 104. Few patients (<1%) had a life impact of little to none at baseline, which increased to 25.0% at week 104. Mean (SD) HIT-6 scores were 65.2 (4.76) at baseline, 57.1 (8.15) at week 4, 56.9 (8.69) at week 48, and 56.1 (9.07) at week 104 (Fig. 5). A clinically meaningful reduction (≥6 points [20]) from baseline in HIT-6 total score was observed at week 4 and sustained or further improved through week 104.

## Discussion

In this analysis, eptinezumab 300 mg, administered once every 12 weeks for up to 8 doses, demonstrated favorable long-term safety and tolerability when used to prevent migraine in patients with CM. A total of 79 (61.7%) patients experienced ≥1 TEAE during the first year of the study and 91 (71.1%) experienced ≥1 TEAE across the entire 2 years, demonstrating a lack of cumulative effect on safety outcomes. Most TEAEs were mild or moderate in severity, the incidence of serious TEAEs and study drug-related AEs was low, and interruption of study drug administration and discontinuation due to TEAEs was infrequent. These findings are consistent with those of double-blind, placebo-controlled trials [11, 12, 23, 24].

Anti-drug antibodies developed in a minority (18%) of patients. The incidence of ADAs was maximal at week 24 (17.5%) and decreased to 5.3% by the end of the primary study phase (week 48) and 0% at the end of the study (week 104), despite continued dosing. This finding is consistent with ADA development in the two phase 3 PROMISE studies, in which ADA prevalence with the 300-mg dose peaked at 17.0–17.5% at week 24 [11, 12]. Overall, only 9/128 patients (7.0%) treated developed NAbs. There was no evidence of an impact from development of ADA, including NAb, on the safety profile of eptinezumab.

Clinically meaningful improvements in PRO measures were observed after the initial eptinezumab dose and were maintained or improved after each subsequent dose. A clinically meaningful change of over twice the American Headache Society threshold [19] in the mean reduction in MIDAS total score was observed at the first assessment (week 12) and sustained through the last assessment (week 104). Similar patterns were observed in the change in self-identified MBS and PGIC measures, with approximately 60% of patients reporting “much improved” or “very much improved” on both measures at week 4 and approximately 80% of patients reporting “much improved” or “very much improved” at week 48. Considering the high level of disability and life-activity interference attributed to migraine, improvements in PROs of this magnitude offer potential markers of clinical benefit [4, 25–28]. In the International Burden of Migraine Study-II (IBMS-II), nearly 85% patients with CM had baseline MIDAS scores indicative of severe disability [4]. In the Chronic Migraine and Epidemiology Outcomes (CaMEO) study, the baseline mean MIDAS score in CM was 60.5 [26]. Thus, improving life impact is a desirable outcome for any migraine intervention.

Persistence with treatment was high in the present study, with 92% of patients completing the first year of the study and 79% completing both years. This represents potential improvement over established oral prophylactic therapies, for which reported persistence rates at 3 months have ranged from 25 to 76% [29]. Data from a retrospective claims analysis suggest that the average time to discontinuation of oral prophylactic therapies is 1–3 months [5]. The most common reasons cited for discontinuation of prophylactic treatment included perceived lack of efficacy (39–48%) and side effects (34–53%) [4]. In the current study, only 8 patients (6.3%) discontinued treatment early because of a TEAE.

The extended duration of PREVAIL permitted evaluation of the safety and tolerability of repeat administration of eptinezumab in the CM population and expanded knowledge of the long-term safety and tolerability of eptinezumab. The 300-mg dose was used to provide safety and tolerability data for the maximum dose level evaluated in pivotal trials. With the exception of patient-identified MBS, the PRO measures included in PREVAIL are recognized by the International Headache Society as valid instruments for assessing patient satisfaction and headache-related healthcare outcomes [30]. To date, this is the longest open-label safety trial for a CGRP monoclonal antibody inhibitor in patients with CM. The lack of a placebo control limits interpretation with regard to clinical relevance and internal validation, especially because placebo responses can be robust in migraine prevention studies [31, 32]. However, the PRO findings in this study were consistent with those from phase 3 pivotal trials.

## Conclusions

In adults with CM, eptinezumab 300 mg administered IV every 12 weeks for up to 84 weeks (8 doses) as a preventive treatment demonstrated a favorable safety and tolerability profile, led to early and sustained reductions in migraine-related burden and improvements in health-related quality of life, and did not result in a discernable impact on the safety profile of patients overall, including those who developed ADA/NAb responses. These findings are in accordance with findings of the PROMISE-2 placebo-controlled pivotal study and provide insight into the long-term safety and tolerability of eptinezumab in the management of patients with CM.

## Supplementary Information

**Additional file 1.** Inclusion and Exclusion Criteria.

## Data Availability

The data reported in this manuscript are part of an ongoing, global sponsor-led clinical development and registration program. Deidentified participant data are not available for legal and ethical reasons.

## Abbreviations

CGRP: Calcitonin gene-related peptide
CM: Chronic migraine
TEAE: Treatment-emergent adverse event
PRO: Patient-reported outcome
ICHD-3β: International Classification of Headache Disorders, 3rd edition, beta
ECG: Electrocardiogram
C-SSRS: Columbia-Suicide Severity Rating Scale
SAE: Serious adverse event
ADA: Anti-drug antibody
MIDAS: Migraine Disability Assessment
MBS: Most bothersome symptom
PGIC: Patient Global Impression of Change
HIT-6: 6-item Headache Impact Test
MedDRA: Medical Dictionary for Regulatory Activities
NAb: Neutralizing antibody
SD: Standard deviation
IBMS-II: International Burden of Migraine Study-II
CaMEO: Chronic Migraine and Epidemiology Outcomes
