# Supplementary material for: Long-term safety and tolerability of eptinezumab in patients with chronic migraine: a 2-year, open-label, phase 3 trial
Source: BMC Neurol. 2021 Mar 19;21:126. doi: 10.1186/s12883-021-02123-w (PMC7977171; doi:10.1186/s12883-021-02123-w)
Supplement: Supplementary file 1 — Additional file 1. Inclusion and Exclusion Criteria. [file 12883_2021_2123_MOESM1_ESM.pdf]

**Long-term safety and tolerability of eptinezumab in patients with chronic migraine: A 2-year, open-label, phase 3 trial**

David Kudrow, MD[1]; Roger K. Cady, MD[2]; Brent Allan, DO[3]; Susan M. Pederson, BS[2]; Joe Hirman, PhD[4]; Lahar R. Mehta, MD[2]; Barbara A. Schaeffler, MBA[2]

Supplemental Materials

Inclusion and Exclusion Criteria

Adults aged 18–65 years (inclusive) with a diagnosis of migraine (ICHD-3 beta, 2013, Section 1.3)[13] at or before the age of 50 years were eligible for participation if they had a history of CM for 12 months or longer prior to screening and had been prescribed or recommended by a healthcare professional to use prescription or over-the-counter medication for acute and/or prophylactic treatment of migraine. Prior migraine prophylactic medication use had to be stable for at least 3 months prior to screening. Hormonal therapy was permitted if it was ongoing for at least 3 months prior to screening. Limited use of barbiturates or prescription opiates by patients was allowed if a stable dose was maintained for 2 months prior to screening and was not expected to exceed 4 days per month during the study. Women of child-bearing potential and men with partners of child-bearing potential were required to agree to use adequate contraception throughout study participation and for at least 6 months after the last dose of study drug.

Individuals were excluded from participation if they had confounding and clinically significant pain syndromes; history of chronic tension-type headache, hypnic headache, cluster headache, hemicrania continua, new daily persistent headache, or sporadic and familial hemiplegic migraine; uncontrolled or untreated psychiatric conditions; had history or diagnosis of a

headache or migraine disorder that did not meet the ICHD-3 beta, 2013, Section 1.3 criteria for CM; had present or previous malignancies; had history or evidence of cardiovascular disease, arteriosclerosis, cardiomyopathy, coronary artery disease, serious heart rhythm abnormalities, neurological disease, cerebrovascular disease, diabetes, Raynaud's disease, or life-threatening allergy; had uncontrolled or newly diagnosed primary or secondary hypertension (mild primary hypertension well controlled for ≥6 months was allowed); had clinically significant abnormal ECG findings; had history or evidence of substance abuse or dependence within the preceding 2 years; had received any experimental, unregistered therapy within 30 days or 5 plasma half-lives before screening; had clinically significant concurrent medical condition or laboratory abnormality at screening or before dosing on day 0; had body mass index ≥39 $kg/m^2$; or had recent or planned surgery requiring general anesthesia within 8 weeks prior to screening or during the duration of the study. Also excluded were patients who had used any prohibited devices, neuromodulation, neurostimulation, or injectable therapy within 2 months prior to screening; botulinum toxin injections for migraine or for any other medical/cosmetic reasons within 4 months prior to screening; any monoclonal antibody targeting the CGRP pathway treatment within 6 months prior to screening; or monoamine oxidase inhibitors, ketamine, methylergonovine, or nimesulide within 3 months prior to screening. Individuals who were at imminent risk of self-harm or harm to others in the investigator's opinion, based on clinical interview and responses provided on the C-SSRS,[14] had a lifetime history of a serious suicide attempt or multiple suicide attempts, any suicidal behavior in the preceding 5 years, or suicidal ideation of type 3, 4, or 5 in the past 6 months were excluded from participation. Women who were pregnant, breastfeeding, or planning to become pregnant during the study were excluded from participation, as were patients who were participating in any other clinical study for the

duration of this trial or within 6 months prior to screening (except clinical studies of CGRP antagonists' last dose >6 months prior to screening with no clinically significant adverse events) or who were positive for HIV, hepatitis B surface antigen, or hepatitis C.
